# Association between consumption of small fish and all-cause mortality among Japanese: the Japan Multi-Institutional Collaborative Cohort Study

**DOI:** 10.1017/S1368980024000831

**Published:** 2024-05-03

**Authors:** Chinatsu Kasahara, Takashi Tamura, Kenji Wakai, Yudai Tamada, Yasufumi Kato, Yoko Kubo, Rieko Okada, Mako Nagayoshi, Asahi Hishida, Nahomi Imaeda, Chiho Goto, Jun Otonari, Hiroaki Ikezaki, Yuichiro Nishida, Chisato Shimanoe, Isao Oze, Yuriko N Koyanagi, Yohko Nakamura, Miho Kusakabe, Daisaku Nishimoto, Ippei Shimoshikiryo, Sadao Suzuki, Miki Watanabe, Etsuko Ozaki, Chie Omichi, Kiyonori Kuriki, Naoyuki Takashima, Naoko Miyagawa, Kokichi Arisawa, Sakurako Katsuura-Kamano, Kenji Takeuchi, Keitaro Matsuo, for the J-MICC Study Group

**Affiliations:** 1 Department of Preventive Medicine, Nagoya University Graduate School of Medicine, Nagoya, Japan; 2 Department of International and Community Oral Health, Tohoku University Graduate School of Dentistry, Sendai, Japan; 3 Department of Public Health, School of Medicine, Aichi Medical University, Nagakute, Japan; 4 Department of Nutrition, Faculty of Wellness, Shigakkan University, Obu, Japan; 5 Department of Public Health, Nagoya City University Graduate School of Medical Sciences, Nagoya, Japan; 6 Department of Health and Nutrition, School of Health and Human Life, Nagoya Bunri University, Inazawa, Japan; 7 Department of Psychosomatic Medicine, Graduate School of Medical Sciences, Kyushu University, Fukuoka, Japan; 8 Department of General Internal Medicine, Kyushu University Hospital, Fukuoka, Japan; 9 Department of Comprehensive General Internal Medicine, Kyushu University Faculty of Medical Sciences, Fukuoka, Japan; 10 Department of Preventive Medicine, Faculty of Medicine, Saga University, Saga, Japan; 11 Department of Pharmacy, Saga University Hospital, Saga, Japan; 12 Division of Cancer Epidemiology and Prevention, Aichi Cancer Center Research Institute, Nagoya, Japan; 13 Cancer Prevention Center, Chiba Cancer Center Research Institute, Chiba, Japan; 14 Department of Epidemiology and Preventive Medicine, Kagoshima University Graduate School of Medical and Dental Sciences, Kagoshima, Japan; 15 School of Health Sciences, Faculty of Medicine, Kagoshima University, Kagoshima, Japan; 16 Environmental Epidemiology Section, Health and Environmental Risk Division, National Institute for Environmental Studies, Tsukuba, Japan; 17 Department of Epidemiology for Community Health and Medicine, Kyoto Prefectural University of Medicine, Kyoto, Japan; 18 Department of Hygiene and Public Health, Osaka Medical and Pharmaceutical University, Takatsuki, Japan; 19 Laboratory of Public Health, Division of Nutritional Sciences, School of Food and Nutritional Sciences, University of Shizuoka, Shizuoka, Japan; 20 Department of Public Health, Shiga University of Medical Science, Otsu, Japan; 21 Department of Preventive Medicine and Public Health, Keio University School of Medicine, Tokyo, Japan; 22 Department of Preventive Medicine, Tokushima University Graduate School of Biomedical Sciences, Tokushima, Japan; 23 Division for Regional Community Development, Liaison Center for Innovative Dentistry, Tohoku University Graduate School of Dentistry, Sendai, Japan; 24 Department of Cancer Epidemiology, Nagoya University Graduate School of Medicine, Nagoya, Japan

**Keywords:** Small fish, All-cause mortality, Cancer, Cohort studies, Japanese

## Abstract

**Objective:**

Although small fish are an important source of micronutrients, the relationship between their intake and mortality remains unclear. This study aimed to clarify the association between intake of small fish and all-cause and cause-specific mortality.

**Design:**

We used the data from a cohort study in Japan. The frequency of the intake of small fish was assessed using a validated FFQ. The hazard ratio (HR) and 95 % confidence interval (CI) for all-cause and cause-specific mortality according to the frequency of the intake of small fish by sex were estimated using a Cox proportional hazard model with adjustments for covariates.

**Setting:**

The Japan Multi-Institutional Collaborative Cohort Study.

**Participants:**

A total of 80 802 participants (34 555 males and 46 247 females), aged 35–69 years.

**Results:**

During a mean follow-up of 9·0 years, we identified 2482 deaths including 1495 cancer-related deaths. The intake of small fish was statistically significantly and inversely associated with the risk of all-cause and cancer mortality in females. The multivariable-adjusted HR (95 % CI) in females for all-cause mortality according to the intake were 0·68 (0·55, 0·85) for intakes 1–3 times/month, 0·72 (0·57, 0·90) for 1–2 times/week and 0·69 (0·54, 0·88) for ≥ 3 times/week, compared with the rare intake. The corresponding HR (95 % CI) in females for cancer mortality were 0·72 (0·54, 0·96), 0·71 (0·53, 0·96) and 0·64 (0·46, 0·89), respectively. No statistically significant association was observed in males.

**Conclusions:**

Intake of small fish may reduce the risk of all-cause and cancer mortality in Japanese females.

## Introduction

Small fish are among the important sources of micronutrients such as Ca, Mg and vitamins A and D when consumed whole with bones and inner organs^(1–6)^. These nutrients contribute to the prevention of non-communicable diseases, including cardiovascular disease (CVD) and cancer, through their antihypertensive, atherosclerosis-inhibiting and antitumour effects^(7–12)^. Bone, eyes and inner organs of fish are reservoirs of most micronutrients, including Ca and vitamin A^(1,2)^. Unlike large fish in which bones and organs are often discarded, small fish offer a unique advantage in that they can be consumed as a whole.

Japanese people habitually eat several types of small fish, including whitebait, Atlantic capelin (shishamo), Japanese smelt (*Hypomesus nipponensis*) (wakasagi), small horse mackerel, young sweetfish and small dried sardine, as a whole. These small fish are consumed in a variety of ways, such as raw or marinated in vinegar, simmered in soy sauce, salted semi-dried and deep-fried. Fish, such as capelin and smelt, are mostly 10–15 cm in length, whereas smaller ones, such as whitebait, are less than 3·5 cm in length^(13)^. These small fish are retailed as frozen or refrigerated products throughout the year. The habit of eating small fish as a whole is also found in other Asian countries besides Japan and some African and European countries. In developing countries, the intake of affordable small fish as a whole is expected to improve severe micronutrient deficiency^(1–3,14)^.

Fish intake has been suggested to be associated with a lower risk of all-cause, cancer and CVD mortality in several cohort studies and meta-analyses, with inconsistent findings for cancer mortality^(15–20)^. Such association, however, has not been specifically assessed for the intake of small fish. Considering that intake of small fish as a whole including the bone and organs may be effective in reducing the mortality risk in a manner different from the ones for non-small fish, it is necessary to assess the association between mortality and the intake of small fish instead of fish consumption in general.

To address this issue, in the present study, we aimed at elucidating the association between the intake of small fish and the risk of all-cause, cancer and CVD mortality using data from a large-scale cohort study in Japan.

## Methods

### Participants

For the present analysis, participants, aged 35–69 years, in the Japan Multi-Institutional Collaborative Cohort (J-MICC) Study were included. The details of the J-MICC Study and the recruitment of participants were described previously^(21)^. In brief, the J-MICC Study is a large cohort study in Japan, which was launched in 2005 and enrolled residents in the community, health check examinees and first-visit patients at a cancer hospital. Some participants were recruited in 2004. The baseline survey included 92 529 adults from 14 study areas (the dataset used in the present study was completed after cleaning the collected data on 1 June 2021). The sample size was determined considering the feasibility including budgets and statistical power for the incidence of major cancer types, which was the primary outcome of the J-MICC Study.

**Fig. 1:**
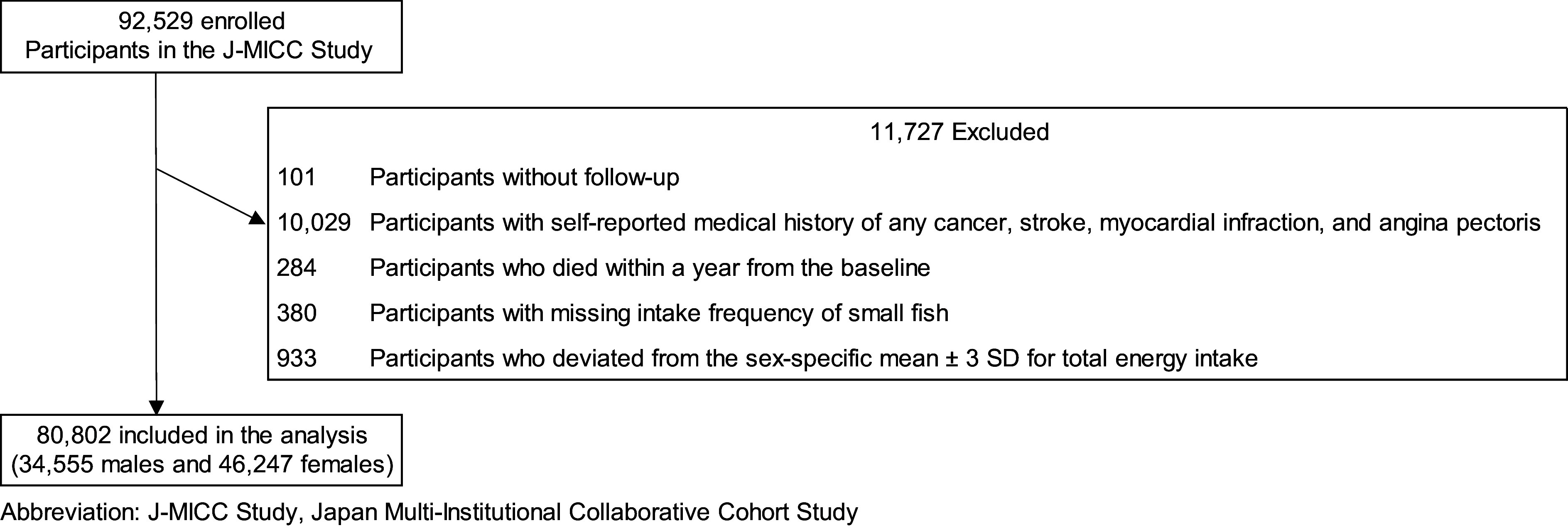
The flow chart for the selection of participants for the present study. (J-MICC Study, Japan Multi-Institutional Collaborative Cohort Study)

Figure 1 shows the flowchart for the selection of participants for the analysis. Participants without a follow-up, those with a self-reported medical history of any cancer, stroke, myocardial infarction and angina pectoris and those who died within a year from the baseline, were excluded. Furthermore, we excluded participants with missing data for the intake of small fish and those who deviated from the sex-specific mean ± 3 sd for total energy intake. Thus, 80 802 individuals (34 555 males and 46 247 females) were finally included in the present study.

### Assessment of lifestyle factors and dietary intake

The height and weight of participants were measured directly on the day of the survey in twelve areas and were self-reported by participants in two areas. BMI was calculated as weight in kilograms divided by the square of height in metres (kg/m^2^). Lifestyle factors, including smoking habit, alcohol consumption, education level, leisure-time physical activity, medical history, age at menarche, number of births and menopausal status were assessed using a self-administered questionnaire at baseline. For smoking habit, participants reported whether they were current smokers, had quit smoking or never smoked. Participants who reported smoking indicated the average number of cigarettes per d, and those who reported quitting indicated how many years (or months) ago they quit. Ethanol intake (g/d) was estimated for current drinkers (defined as those who consumed alcohol at least once a month during the last year) based on the reported consumption frequency and amount consumed each time for six alcoholic beverages (Japanese *sake*, *shochu*, *shochu*-based cocktails, beer, whisky and wine)^(22)^. For education level, participants reported the last school level they graduated from (excluding dropout) from one of the following seven categories: elementary school or junior high school, high school, vocational school, junior college or technical school, college or university, graduate school and others. Participants from three study areas were not asked about their education level and were assigned to an additional category for missing data. Leisure-time physical activity was estimated based on the frequency and duration of leisure-time activities^(23)^. Leisure-time physical activity was calculated as metabolic equivalent hours per d (MET·h/d) by multiplying the assigned daily mean frequency, mean duration (time in hours) and MET value together for each activity: low-intensity physical activity (e.g. walking and golf, assigned 3·4 MET), moderate-intensity physical activity (e.g. jogging and swimming, 7·0 MET) and high-intensity physical activity (e.g. marathon running and martial arts, 10 MET). The frequency (assigned as daily mean frequency) was reported in five categories as follows: none (0), 1–3 times/month (2/30), 1–2 times/week (1·5/7), 3–4 times/week (3·5/7) and ≥ 5 times/week (6/7). The mean duration (assigned as time in hours) was reported in six categories as follows: < 30 min (15/60), 30 min to < 1 h (45/60), 1 to < 2 h (1·5), 2 to < 3 h (2·5), 3 to < 4 h (3·5) and ≥ 4 h (4·0). The questionnaire was based on a similar validated survey used in the Japan Public Health Center-based Prospective Study^(24)^. The patient’s medical history was self-reported, and the past and present history were considered a positive history. Age at menarche, number of births and menopausal status were also self-reported.

The average daily intake of energy, selected foods/food groups (green and yellow vegetables, light-coloured vegetables, fruit, meat and rice) and nutrients (Na, dietary fibre, *n*-3 HUFA (including EPA, DHA and docosapentaenoic acid), Ca, vitamin D, retinol and carotene) were estimated using a validated short FFQ, including forty-seven foods and beverages, based on the Standard Tables of Food Composition in Japan, the fifth revised edition at baseline^(25–28)^. Retinol intake was estimated as retinol equivalents, and carotene intake was estimated as *β*-carotene equivalents^(28)^. Only the baseline FFQ results were considered. Nutrient intakes from supplements were not included. The FFQ included seven questions that assessed the intake of fish (raw fish, grilled fish and boiled fish), small fish (Atlantic capelin and dried young sardines (whitebait)), canned tuna, crustacean and molluscs (shrimp, crab, octopus and squid), shellfish (clams and oysters), roe (salted cod roe and salmon roe) and fish-paste products (baked bar (chikuwa) and steamed cake (kamaboko)) with eight possible responses on intake frequency (rarely, 1–3 times/month, 1–2 times/week, 3–4 times/week, 5–6 times/week, 1 time/d, 2 times/d and ≥ 3 times/d). The items listed in parentheses in the FFQ are examples of questions preceding the parentheses. Atlantic capelin and dried young sardines (whitebait) were listed as examples of small fish in the parentheses. We evaluated the validity of the intake of small fish estimated using the FFQ by comparing the estimate with the intake based on 12-d dietary records. Both the intakes were log_e_-transformed. The Pearson’s correlation coefficients (de-attenuated for intra-individual variation) between the FFQ and dietary records were 0·48 for males and 0·51 for females^(27)^. We adjusted for total energy from the intake of foods and nutrients using the density method^(29)^. We then divided the subjects into sex-specific quartiles according to energy-adjusted intake of each food or nutrient. To validate the consumption of other foods as an adjusted covariate, the Spearman’s correlation coefficients (de-attenuated for intra-individual variation and energy-adjusted) between the FFQ and dietary records were calculated and determined to be 0·34 in males and 0·36 in females for green and yellow vegetables, 0·35 in males and 0·24 in females for light-coloured vegetables, 0·62 in males and 0·58 in females for fruit, 0·41 in males and 0·41 in females for meat and 0·67 in males and 0·61 in females for rice^(27)^.

Regarding the total energy and nutrient intake adjusted as covariates, the Pearson’s correlation coefficients (de-attenuated for intra-individual variation, log_e_-transformed and energy-adjusted) between the FFQ and dietary records were determined as 0·49 in males and 0·44 in females for total energy, 0·24 in males and 0·35 in females for Na, 0·36 in males and 0·47 in females for dietary fibre, 0·36 in males and 0·35 in females for *n*-3 HUFA, 0·49 in males and 0·59 in females for Ca, 0·65 in males and 0·40 in females for vitamin D, 0·27 in males and 0·22 in females for retinol and 0·39 in males and 0·38 in females for carotene^(26)^.

We calculated the Japanese diet index (JDI) to examine whether the intake of small fish is associated with mortality risk, independently of the degree of adherence to the Japanese diet. This is because the Japanese diet is reported to be associated with a lower risk of mortality^(30,31)^. The original JDI consists of the following eight components: rice, miso soup, seaweeds, pickles, green and yellow vegetables, fish, green tea and beef and pork. We used only seven factors, excluding pickles, for the JDI because of no information on pickles. Pickles are important Na sources in the Japanese diet, so they are sometimes incorporated in an FFQ to estimate Na intake. In the development of the FFQ used in our study, however, pickles were not included because Na intake was not an original target nutrient. The method to estimate Na intake was devised after FFQ development. Since the FFQ is reasonably valid for small fish intake, it was appropriate for the present study hypothesis. The fish component of the JDI was calculated using the total consumption of fish and shellfish, including small fish. The beef and pork represent non-adherence to the Japanese diet, and participants received one point if their intake (g/d) was less than the sex-specific median for the entire population of this study. The remaining six components represent adherence to the Japanese diet; participants received one point if the intake (g/d) was more than or equal to the sex-specific median for the entire population of this study. The intakes of seven components for participants with missing data for the intake were considered zero. The JDI score ranged from 0 to 7, with higher scores indicating greater conformity to the Japanese diet.

### Follow-up and endpoint

We followed eligible participants from the enrolment date (from 11 February 2004 to 31 March 2014) to 31 December 2017 in eleven study areas (Chiba, Aichi Cancer Center, Okazaki, Shizuoka, Takashima, Kyoto, Fukuoka, Saga, Kagoshima, Tokushima and Shizuoka-Sakuragaoka) or to 31 December 2018 in three ones (Kyushu and Okinawa Population Study (KOPS), Iga and Daiko). Information on residence and survival status was obtained from the resident registers annually or biennially. During the follow-up period, 4400 participants (5·4 %) were moved out of the study areas, and 165 participants (0·2 %) were unable to follow up because of other reasons. They were censored at the last date when they were known to reside in the study areas. The causes of death were identified based on the abstracts of death certificates provided by the Japanese Ministry of Health, Labour and Welfare and were coded according to the International Classification of Diseases and Related Health Problems, Tenth Revision (ICD-10). The primary outcome of this study was death of all causes, and the secondary outcomes were death of cancer (ICD-10: C00–C97), CVD including heart disease and cerebrovascular disease (ICD-10: I00–I99) and other causes (non-cancer, non-CVD).

### Statistical analysis

Participants were classified into four groups according to the frequency of the intake of small fish by sex, as follows: rarely, 1–3 times/month, 1–2 times/week and ≥ 3 times/week. Differences in the means of age and leisure-time physical activity between the frequency categories were tested using one-way ANOVA. Differences in the median of energy-adjusted intakes of small fish and non-small fish (raw fish, grilled fish and boiled fish; do not include other seafood items) between the frequency categories were analysed using the Kruskal-Wallis test. Differences in the proportions of categorical variables between the frequency groups were tested using the *χ*^2^ test. Because a considerable proportion of participants (40·8 %) filled out the FFQ and questionnaire for lifestyle factors only at baseline, only baseline responses to the FFQ and questionnaire were considered, as these were provided by all the participants.

The sex-specific hazard ratio (HR) and corresponding 95 % confidence interval (CI) for all-cause, cancer, CVD and other-cause mortality according to the frequency of the intake of small fish were estimated using the Cox proportional hazards model with adjustments for potential confounding factors. The end of follow-up was defined as the date of death, the date of moving out of the study area or the end of follow-up, whichever occurred first. The lowest category of the intake of small fish (rarely) was considered the reference group. The categories were determined so that each category has enough number of participants. The linear trends for the risk were evaluated using an ordinal number assigned to the frequency categories (rarely: 1; 1–3 times/month: 2; 1–2 times/week: 3; and ≥ 3 times/week: 4).

Four multivariable models were established as follows: Model 1: adjusted for age at baseline (as a continuous variable) and study areas (Chiba, Aichi Cancer Center, Okazaki, Shizuoka, Iga, Daiko, Takashima, Kyoto, Fukuoka, Saga, Kagoshima, Tokushima, Kyushu and Okinawa Population Study (KOPS), Shizuoka-Sakuragaoka). Model 2: adjusted for covariates in Model 1 plus BMI (< 18·5, 18·5 to < 25, ≥ 25 kg/m^2^), smoking habit (for males: never, former (quit smoking ≥ 10, 5 to < 10, < 5 years ago), current (< 20, 20–< 40, ≥ 40 cigarettes/d); for females: never, former (quit smoking ≥ 10, < 10 years ago), current (< 20, ≥ 20 cigarettes/d)), alcohol consumption (for males: never, former, current (< 23, 23 to < 46, ≥ 46 g/d ethanol); for females: never, former, current (< 23, ≥ 23 g/d ethanol); 23 g/d ethanol is equivalent to 180 mL of Japanese *sake*), education level (junior high school or under (≤ 9 years), high school (10–12 years), junior college or vocational school (13–15 years) and college, university or above (≥ 16 years)), leisure-time physical activity (MET·h/d; as a continuous variable) and self-reported medical history of hypertension, diabetes and dyslipidaemia (yes or no) for males; for females, the same covariates were used as in males, plus age at menarche (≤ 12, 13, 14, ≥ 15 years), number of births (none, 1, 2, 3, ≥ 4) and menopausal status (pre-menopausal, menopausal (age at menopause < 47, 47 to < 50, 50 to < 53, ≥ 53 years old)). Model 3: adjusted for covariates in Model 2 plus total energy intake (by quartile) and energy-adjusted intakes of green and yellow vegetables, light-coloured vegetables, fruit, meat, rice, Na and dietary fibre (by quartile) and JDI score (points of 0–1, 2, 3, 4, 5, 6–7). For Model 4, we additionally adjusted for intakes of nutrients abundant in small fish, including *n*-3 HUFA, Ca, vitamin D, retinol and carotene (by quartile) which might mediate the association between small fish and mortality. We did not conduct a formal mediation analysis. We consider Model 3 to represent the main result in the present study. We confirmed the proportional hazards assumption in Model 3 by including each frequency category for the intake of small fish (1–3 times/month, 1–2 times/week and ≥ 3 times/week) × time (continuous) interaction terms. The assumption was not violated (*P* > 0·05) except for the interaction term for the small fish ≥ 3 times/week group × time in the analysis of male cancer mortality (*P* = 0·04). We also assessed the interaction between sex and intake of small fish on the mortality risk with Model 3 including the cross-product term (i.e. sex (dichotomous) × category of small fish consumed (continuous)), representing the interaction. The adjustment variables in Model 3 were different between sexes. Thus, in this analysis, we excluded the female-specific variables (age at menarche, number of births and menopausal status) and adjusted for smoking habit and alcohol consumption to the same category used in females (smoking habit: never, former (quit smoking ≥ 10, < 10 years ago), current (< 20, ≥ 20 cigarettes/d)); alcohol consumption: never, former, current (< 23, ≥ 23 g/d ethanol)). The total energy intake and the intakes of food groups and nutrients were adjusted for sex-specific quartiles. The JDI was calculated with the sex-specific median for the entire population of this study.

We analysed 80 250 participants (34 169 males and 46 081 females), additionally excluding 552 participants who died 1–3 years after baseline measurements, using the same covariates as in Model 3. The participants who died within 1 year had been already excluded (Fig. 1). This analysis was added to consider the influence of diseases that might have existed at baseline over a longer period than that considered in Model 3 (3 years *v*. 1 year). We further analysed 75 121 participants (32 316 males and 42 805 females), excluding 5681 participants from the Aichi Cancer Center using the same covariates as in Model 3 because they might have included undiagnosed cancer patients at baseline. Additionally, we performed stratified analyses by age (≥ 60, < 60 years old), smoking status (never, (former or current)) and JDI score (≤ 3, ≥ 4 points), with adjustment for the same covariates as in Model 3. Effects of interactions between stratification variables and intake of small fish on the mortality risk were assessed with Model 3 including the cross-product term (i.e. stratification factors (dichotomous) × category of small fish consumed (continuous)), representing the interaction. Lastly, we considered the intake of non-small fish (raw fish, grilled fish and boiled fish; do not include other seafood items) for the association of the intake of small fish with all-cause, cancer, CVD and other-cause mortality using the same covariates as in Model 3 by further adjustment for the frequency of the intake of non-small fish (≤ 2 times/week, 3–4 times/week, 5–6 times/week and ≥ 1 time/d), excluding 115 participants (34 males and 81 females) with missing data for the intake of non-small fish.

Participants with missing data for covariates were included as additional categories in the analysis. A two-tailed *P* value < 0·05 was considered statistically significant. All statistical analyses were conducted using the SPSS software, version 28 (IBM) and Stata/SE17 (StataCorp).

## Results

### Characteristics of participants

Tables 1 and 2 present the baseline characteristics according to the frequency of the intake of small fish in males and females, respectively. The mean age (sd) of 80 802 eligible participants (34 555 males and 46 247 females) was 54·7 (9·4) years. Those with frequent intake of small fish were more likely to be aged, non-lean, non-smoker (never or former smoker), current drinker (in males), physically active and having hypertension and menopausal (in females). The distribution of education level and study area differed statistically significantly according to the frequency of the intake of small fish. The intake of small fish was positively correlated with the intake of all nutrients and foods (except for rice in males), total energy and the JDI score.

**Table 1:** Baseline characteristics in males according to the frequency of the intake of small fish*

	Rarely		1–3 times/month		1–2 times/week		≥ 3 times/week		Total		
	*n*	%	*n*	%	*n*	%	*n*	%	*n*	%	*P* value
Participants, *n*	5046		14 579		9890		5040		34 555		
Age, years											
Mean	52·8		53·9		56·5		59·4		55·3		< 0·001
sd	9·6		9·4		8·9		8·2		9·4		
Study area											
Kanto (Chiba)	373	7·4	1227	8·4	683	6·9	292	5·8	2575	7·5	< 0·001
Chubu (Aichi Cancer Center, Okazaki, Shizuoka, Iga, Daiko, Shizuoka-Sakuragaoka)	1573	31·2	5553	38·1	4050	41·0	1886	37·4	13 062	37·8	
Kinki (Takashima, Kyoto)	485	9·6	1389	9·5	973	9·8	540	10·7	3387	9·8	
Shikoku (Tokushima)	215	4·3	596	4·1	347	3·5	144	2·9	1302	3·8	
Kyushu and Okinawa (Fukuoka, Saga, Kagoshima, KOPS)	2400	47·6	5814	39·9	3837	38·8	2178	43·2	14 229	41·2	
BMI, kg/m^2^											
18·5–24·9	3238	64·2	9803	67·2	6730	68·0	3394	67·3	23 165	67·0	< 0·001
< 18·5	168	3·3	377	2·6	241	2·4	104	2·1	890	2·6	
≥ 25·0	1636	32·4	4390	30·1	2916	29·5	1538	30·5	10 480	30·3	
Unknown	4	0·1	9	0·1	3	0·0	4	0·1	20	0·1	
Smoking habit											
Never	1522	30·2	4188	28·7	2883	29·2	1562	31·0	10 155	29·4	< 0·001
Former, quit ≥ 10·0 years before	916	18·2	3126	21·4	2375	24·0	1302	25·8	7719	22·3	
Former, quit 5·0–9·9 years before	256	5·1	941	6·5	615	6·2	321	6·4	2133	6·2	
Former, quit < 5·0 years before	458	9·1	1373	9·4	884	8·9	434	8·6	3149	9·1	
Current, < 20 cigarettes/d	511	10·1	1420	9·7	933	9·4	421	8·4	3285	9·5	
Current, 20–39 cigarettes/d	1051	20·8	2751	18·9	1723	17·4	739	14·7	6264	18·1	
Current, ≥ 40 cigarettes/d	205	4·1	424	2·9	222	2·2	121	2·4	972	2·8	
Unknown	127	2·5	356	2·4	255	2·6	140	2·8	878	2·5	
Alcohol consumption											
Never	1378	27·3	2948	20·2	1822	18·4	838	16·6	6986	20·2	< 0·001
Former	209	4·1	397	2·7	256	2·6	183	3·6	1045	3·0	
Current, < 23·0 g/d ethanol	1678	33·3	5420	37·2	3560	36·0	1692	33·6	12 350	35·7	
Current, 23·0–45·9 g/d ethanol	865	17·1	2977	20·4	2122	21·5	1165	23·1	7129	20·6	
Current, ≥ 46·0 g/d ethanol	910	18·0	2821	19·3	2120	21·4	1157	23·0	7008	20·3	
Unknown	6	0·1	16	0·1	10	0·1	5	0·1	37	0·1	
Education level											
Junior high school (≤ 9 years)	380	7·5	869	6·0	767	7·8	575	11·4	2591	7·5	< 0·001
High school (10–12 years)	1550	30·7	4372	30·0	2932	29·6	1425	28·3	10 279	29·7	
Junior college or vocational school (13–15 years)	478	9·5	1338	9·2	813	8·2	390	7·7	3019	8·7	
College, university or above (≥ 16 years)	1447	28·7	5087	34·9	3213	32·5	1304	25·9	11 051	32·0	
No question in the questionnaire	1176	23·3	2848	19·5	2127	21·5	1319	26·2	7470	21·6	
Others or unknown	15	0·3	65	0·4	38	0·4	27	0·5	145	0·4	
Leisure-time physical activity											
MET·h/d											
Mean	1·6		2·0		2·4		2·7		2·1		< 0·001
sd	2·9		3·1		3·6		3·8		3·4		
Hypertension											
No	4034	79·9	11 522	79·0	7565	76·5	3698	73·4	26 819	77·6	< 0·001
Yes	998	19·8	3024	20·7	2304	23·3	1334	26·5	7660	22·2	
Unknown	14	0·3	33	0·2	21	0·2	8	0·2	76	0·2	
Diabetes											
No	4633	91·8	13 484	92·5	9000	91·0	4523	89·7	31 640	91·6	< 0·001
Yes	401	7·9	1069	7·3	875	8·8	506	10·0	2851	8·3	
Unknown	12	0·2	26	0·2	15	0·2	11	0·2	64	0·2	
Dyslipidaemia											
No	4278	84·8	12 371	84·9	8321	84·1	4309	85·5	29 279	84·7	0·452
Yes	752	14·9	2154	14·8	1535	15·5	713	14·1	5154	14·9	
Unknown	16	0·3	54	0·4	34	0·3	18	0·4	122	0·4	
	Median	IQR	Median	IQR	Median	IQR	Median	IQR	Median	IQR	
Small fish intake†, g/1000 kcal/d	0·0	0–0	1·1	1·0–1·2	2·1	1·9–2·3	5·7	4·9–8·4	1·3	1·0–2·2	< 0·001
Non-small fish intake†,‡, g/1000 kcal/d	8·1	5·5–17·9	8·9	7·1–19·0	16·3	7·7–20·5	19·7	15·7–29·0	13·8	7·3–20·3	< 0·001
Energy intake, kcal/d	1813·7	1613·7–2021·3	1849·8	1673·7–2050·4	1911·2	1741·7–2103·7	1961·6	1787·4–2164·8	1880·6	1697·4–2081·4	< 0·001
Rice intake†, g/1000 kcal/d	229·6	180·1–278·5	228·3	182·7–274·4	234·2	188·3–278·3	238·3	191·5–278·7	231·3	185·3–276·9	< 0·001
Meat intake†, g/1000 kcal/d	15·9	10·9–24·1	16·2	11·9–24·3	16·7	12·8–24·8	17·6	12·4–27·2	16·5	12·1–24·8	< 0·001
Fruits intake†, g/1000 kcal/d	10·3	4·8–20·7	13·7	8·8–27·8	17·5	10·1–36·3	25·1	12·3–47·2	15·4	9·0–33·0	< 0·001
Green and yellow vegetables intake†, g/1000 kcal/d	18·3	11·4–30·3	21·9	15·0–34·3	27·5	18·6–41·1	35·6	22·1–53·1	24·4	16·0–38·7	< 0·001
Light-coloured vegetables intake†, g/1000 kcal/d	20·4	12·6–32·3	22·8	14·6–33·8	27·2	17·5–38·8	34·7	23·4–48·0	25·2	15·7–37·4	< 0·001
Na intake, mg/1000 kcal/d	862·4	705·7–1060·3	893·6	749·3–1073·1	922·5	777·6–1109·3	1024·3	865·1–1235·8	916·4	765·3–1105·8	< 0·001
Dietary fibre intake, g/1000 kcal/d	4·7	3·9–5·6	4·9	4·2–5·8	5·2	4·4–6·2	5·9	4·8–7·0	5·1	4·3–6·1	< 0·001
Ca intake, mg/1000 kcal/d	226·3	185·7–276·6	236·6	197·9–287·3	249·1	208·0–302·6	284·2	235·2–344·1	245·2	202·8–299·7	< 0·001
Vitamin D intake, μg/1000 kcal/d	2·6	2·0–3·7	3·0	2·5–4·2	3·9	2·9–4·8	5·6	4·6–7·0	3·6	2·6–4·8	< 0·001
*n*-3 HUFA intake, mg/1000 kcal/d	269·7	216·3–400·0	309·6	250·2–438·2	394·4	283·9–486·9	516·2	421·7–655·8	361·8	262·4–484·6	< 0·001
Retinol intake, μg/1000 kcal/d	343·8	276·2–489·0	428·4	308·4–585·9	491·1	342·3–638·9	537·2	380·4–703·3	447·0	315·6–608·5	< 0·001
Carotene intake, μg/1000 kcal/d	1199·7	962·6–1574·6	1267·3	1036·9–1642·7	1406·8	1116·3–1825·9	1624·3	1218·8–2127·9	1336·3	1061·0–1764·9	< 0·001
JDI score, points											
0–1	1245	24·7	2222	15·2	603	6·1	68	1·3	4138	12·0	< 0·001
2	1157	22·9	2787	19·1	1054	10·7	252	5·0	5250	15·2	
3	1054	20·9	3316	22·7	1853	18·7	586	11·6	6809	19·7	
4	872	17·3	3079	21·1	2448	24·8	1086	21·5	7485	21·7	
5	507	10·0	2141	14·7	2428	24·6	1542	30·6	6618	19·2	
6–7	211	4·2	1034	7·1	1504	15·2	1506	29·9	4255	12·3	

IQR, interquartile range; JDI, Japanese diet index; KOPS, Kyushu and Okinawa Population Study; MET, metabolic equivalent.

* Values are numbers (percentages) unless indicated otherwise.

† Food consumption of the total population including non-consumers was used.

‡ Excluded thirty-four males with missing data for the intake of non-small fish.

**Table 2:** Baseline characteristics in females according to the frequency of the intake of small fish*

	Rarely		1–3 times/month		1–2 times/week		≥ 3 times/week		Total		
	*n*	%	*n*	%	*n*	%	*n*	%	*n*	%	*P* value
Participants, *n*	5599		17 562		13 746		9340		46 247		
Age, years											
Mean	51·0		52·2		55·2		58·7		54·3		< 0·001
sd	9·6		9·2		9·1		8·2		9·4		
Study area											
Kanto (Chiba)	537	9·6	1962	11·2	1422	10·3	822	8·8	4743	10·3	< 0·001
Chubu (Aichi Cancer Center, Okazaki, Shizuoka, Iga, Daiko, Shizuoka-Sakuragaoka)	1572	28·1	5563	31·7	4397	32·0	2516	26·9	14 048	30·4	
Kinki (Takashima, Kyoto)	520	9·3	1957	11·1	1707	12·4	1190	12·7	5374	11·6	
Shikoku (Tokushima)	174	3·1	362	2·1	260	1·9	134	1·4	930	2·0	
Kyushu and Okinawa (Fukuoka, Saga, Kagoshima, KOPS)	2796	49·9	7718	43·9	5960	43·4	4678	50·1	21 152	45·7	
BMI, kg/m^2^											
18·5–24·9	3930	70·2	12 810	72·9	10 089	73·4	6651	71·2	33 480	72·4	< 0·001
< 18·5	587	10·5	1649	9·4	1234	9·0	787	8·4	4257	9·2	
≥ 25·0	1070	19·1	3078	17·5	2400	17·5	1893	20·3	8441	18·3	
Unknown	12	0·2	25	0·1	23	0·2	9	0·1	69	0·1	
Smoking habit											
Never	4397	78·5	14 577	83·0	11 978	87·1	8414	90·1	39 366	85·1	< 0·001
Former, quit ≥ 10·0 years before	206	3·7	652	3·7	423	3·1	235	2·5	1516	3·3	
Former, quit < 10·0 years before	273	4·9	696	4·0	413	3·0	204	2·2	1586	3·4	
Current, < 20 cigarettes/d	395	7·1	976	5·6	523	3·8	258	2·8	2152	4·7	
Current, ≥ 20 cigarettes/d	253	4·5	468	2·7	265	1·9	144	1·5	1130	2·4	
Unknown	75	1·3	193	1·1	144	1·0	85	0·9	497	1·1	
Alcohol consumption											
Never	3423	61·1	10 118	57·6	8359	60·8	6107	65·4	28 007	60·6	< 0·001
Former	142	2·5	328	1·9	206	1·5	158	1·7	834	1·8	
Current, < 23·0 g/d ethanol	1666	29·8	6040	34·4	4488	32·6	2611	28·0	14 805	32·0	
Current, ≥ 23·0 g/d ethanol	356	6·4	1057	6·0	672	4·9	450	4·8	2535	5·5	
Unknown	12	0·2	19	0·1	21	0·2	14	0·1	66	0·1	
Education level											
Junior high school (≤ 9 years)	331	5·9	911	5·2	878	6·4	888	9·5	3008	6·5	< 0·001
High school (10–12 years)	1776	31·7	5809	33·1	4571	33·3	3002	32·1	15 158	32·8	
Junior college or vocational school (13–15 years)	1373	24·5	4999	28·5	3440	25·0	1978	21·2	11 790	25·5	
College, university or above (≥ 16 years)	535	9·6	1993	11·3	1461	10·6	738	7·9	4727	10·2	
No question in the questionnaire	1564	27·9	3792	21·6	3337	24·3	2687	28·8	11 380	24·6	
Others or unknown	20	0·4	58	0·3	59	0·4	47	0·5	184	0·4	
Leisure-time physical activity											
MET·h/d											
Mean	1·3		1·6		2·0		2·4		1·9		< 0·001
sd	2·5		2·7		3·0		3·4		2·9		
Hypertension											
No	4928	88·0	15 302	87·1	11 527	83·9	7604	81·4	39 361	85·1	< 0·001
Yes	657	11·7	2219	12·6	2197	16·0	1711	18·3	6784	14·7	
Unknown	14	0·3	41	0·2	22	0·2	25	0·3	102	0·2	
Diabetes											
No	5418	96·8	17 057	97·1	13 304	96·8	8952	95·8	44 731	96·7	< 0·001
Yes	166	3·0	476	2·7	424	3·1	366	3·9	1432	3·1	
Unknown	15	0·3	29	0·2	18	0·1	22	0·2	84	0·2	
Dyslipidaemia											
No	4881	87·2	15 178	86·4	11 590	84·3	7723	82·7	39 372	85·1	< 0·001
Yes	698	12·5	2332	13·3	2107	15·3	1580	16·9	6717	14·5	
Unknown	20	0·4	52	0·3	49	0·4	37	0·4	158	0·3	
Age at menarche, years											
≤ 12	1977	35·3	6432	36·6	4221	30·7	2345	25·1	14 975	32·4	< 0·001
13	1368	24·4	4450	25·3	3478	25·3	2278	24·4	11 574	25·0	
14	1259	22·5	4015	22·9	3464	25·2	2467	26·4	11 205	24·2	
≥ 15	940	16·8	2579	14·7	2501	18·2	2202	23·6	8222	17·8	
Unknown	55	1·0	86	0·5	82	0·6	48	0·5	271	0·6	
Number of births											
None	697	12·4	1528	8·7	864	6·3	594	6·4	3683	8·0	< 0·001
1	653	11·7	2030	11·6	1423	10·4	879	9·4	4985	10·8	
2	2091	37·3	7669	43·7	6258	45·5	4200	45·0	20 218	43·7	
3	1193	21·3	4282	24·4	3677	26·7	2596	27·8	11 748	25·4	
≥ 4	473	8·4	1084	6·2	944	6·9	739	7·9	3240	7·0	
Unknown	492	8·8	969	5·5	580	4·2	332	3·6	2373	5·1	
Menopausal status											
Pre-menopausal	2823	50·4	8104	46·1	4447	32·4	1740	18·6	17 114	37·0	< 0·001
Menopause at < 47 years old	592	10·6	1689	9·6	1524	11·1	1203	12·9	5008	10·8	
Menopause at 47–< 50 years old	516	9·2	1639	9·3	1525	11·1	1204	12·9	4884	10·6	
Menopause at 50–< 53 years old	1012	18·1	3732	21·3	3753	27·3	3031	32·5	11 528	24·9	
Menopause at ≥ 53 years old	593	10·6	2242	12·8	2352	17·1	2045	21·9	7232	15·6	
Unknown	63	1·1	156	0·9	145	1·1	117	1·3	481	1·0	
	Median	IQR	Median	IQR	Median	IQR	Median	IQR	Median	IQR	
Small fish intake†, g/1000 kcal/d	0·0	0–0	1·0	0·9–1·1	1·9	1·8–2·1	5·6	4·7–8·4	1·5	0·9–2·2	< 0·001
Non-small fish intake†,‡, g/1000 kcal/d	8·6	6·7–19·2	10·9	7·8–20·4	18·3	8·5–22·5	20·4	17·0–30·7	17·7	8·1–22·4	< 0·001
Energy intake, kcal/d	1478·4	1322·2–1613·0	1512·3	1375·0–1636·4	1549·3	1416·8–1673·2	1591·1	1457·6–1718·7	1535·0	1394·7–1664·8	< 0·001
Rice intake†, g/1000 kcal/d	176·1	128·5–217·8	177·1	134·8–218·3	182·1	141·3–224·9	192·1	150·1–235·1	181·6	138·8–224·1	< 0·001
Meat intake†, g/1000 kcal/d	22·1	15·2–32·7	23·0	16·5–33·1	23·8	16·8–34·1	24·2	16·0–35·5	23·3	16·4–33·9	< 0·001
Fruits intake†, g/1000 kcal/d	20·3	10·9–49·1	25·5	14·0–56·1	39·2	20·1–66·5	53·0	23·4–82·8	35·1	16·5–63·9	< 0·001
Green and yellow vegetables intake†, g/1000 kcal/d	33·4	21·7–52·3	38·6	25·9–56·6	47·6	32·7–67·7	59·3	41·3–82·2	44·5	29·0–65·5	< 0·001
Light-coloured vegetables intake†, g/1000 kcal/d	37·6	24·6–53·1	41·1	28·5–56·2	46·8	33·8–62·8	55·7	41·1–73·7	45·2	31·3–61·7	< 0·001
Na intake, mg/1000 kcal/d	1029·2	866·8–1213·5	1068·5	919·2–1243·1	1105·5	961·6–1280·6	1205·6	1042·2–1408·8	1102·5	946·7–1287·0	< 0·001
Dietary fibre intake, g/1000 kcal/d	6·5	5·6–7·7	6·8	5·9–8·0	7·4	6·4–8·7	8·3	7·0–9·7	7·2	6·1–8·5	< 0·001
Ca intake, mg/1000 kcal/d	312·2	254·5–378·8	326·0	271·8–389·6	342·9	290·6–406·7	381·6	323·5–452·1	340·7	283·9–407·7	< 0·001
Vitamin D intake, μg/1000 kcal/d	3·0	2·5–4·3	3·8	3·0–4·8	4·6	3·5–5·5	6·2	5·2–7·6	4·5	3·2–5·7	< 0·001
*n*-3 HUFA intake, mg/1000 kcal/d	313·5	258·1–453·7	386·3	294·8–495·4	460·3	338·5–555·1	563·7	470·4–712·7	446·2	313·4–559·3	< 0·001
Retinol intake, μg/1000 kcal/d	484·6	380·1–660·4	560·2	421·3–761·0	640·1	469·5–834·9	710·4	525·1–922·6	602·3	444·0–808·8	< 0·001
Carotene intake, μg/1000 kcal/d	1829·0	1419·8–2421·8	1952·6	1531·6–2492·0	2180·5	1714·6–2766·8	2510·6	1965·1–3166·5	2114·9	1628·8–2720·8	< 0·001
JDI score, points											
0–1	1368	24·4	2431	13·8	669	4·9	116	1·2	4584	9·9	< 0·001
2	1219	21·8	3264	18·6	1521	11·1	343	3·7	6347	13·7	
3	1233	22·0	4185	23·8	2655	19·3	963	10·3	9036	19·5	
4	958	17·1	3927	22·4	3624	26·4	2013	21·6	10 522	22·8	
5	576	10·3	2624	14·9	3323	24·2	3021	32·3	9544	20·6	
6–7	245	4·4	1131	6·4	1954	14·2	2884	30·9	6214	13·4	

IQR, interquartile range; JDI, Japanese diet index; KOPS, Kyushu and Okinawa Population Study; MET, metabolic equivalent.

* Values are numbers (percentages) unless indicated otherwise.

† Food consumption of the total population including non-consumers was used.

‡ Excluded eighty-one females with missing data for the intake of non-small fish.

### All-cause mortality

During the 724 115 person-year follow-up (mean, 9·0 years), we identified 2482 deaths (1618 in males and 864 in females), including 1495 cancer-related deaths (988 in males and 507 in females), 340 CVD deaths (204 in males and 136 in females) and 647 other-cause deaths (426 in males and 221 in females). In males, the top five sites for all cancer-related deaths were the lung (25·1 %), pancreas (12·2 %), stomach (10·9 %), esophagus (9·3 %) and colorectum (9·2 %). In females, the top five sites were the lung (16·6 %), pancreas (12·2 %), colorectum (11·8 %), breast (8·7 %) and stomach (7·1 %).

The association between the frequency of the intake of small fish and the risk of all-cause mortality by sex is shown in Table 3. In Models 1 and 2, with adjustment for multiple covariates, the intake of small fish was inversely associated with the risk of all-cause mortality in both sexes. In Model 3, with further adjustment for energy-adjusted intake of foods and nutrients and JDI score, the inverse association remained statistically significant in females, but not in males. The multivariable-adjusted HR (95 % CI) in females were 0·68 (0·55, 0·85) for intakes 1–3 times/month, 0·72 (0·57, 0·90) for 1–2 times/week and 0·69 (0·54, 0·88) for ≥ 3 times/week, compared with the rare intake (*P*_for trend_ = 0·041). The corresponding HR (95 % CI) in males were 0·81 (0·69, 0·94), 0·84 (0·71, 0·99) and 0·87 (0·73, 1·05), respectively (*P*_for trend_ = 0·391). In Model 4, with an additional adjustment for the energy-adjusted intake of nutrients abundant in small fish, the inverse association between the intake of small fish and all-cause mortality was observed in females although the HR for intake ≥ 3 times/week was higher than that in Model 3. No statistically significant association was observed in males in Model 4.

**Table 3:** Multivariable-adjusted HR (95 % CI) for all-cause, cancer, CVD and other-cause mortality by sex according to the frequency of the intake of small fish

	Male								Female								
		1–3 times/month		1–2 times/week		≥ 3 times/week				1–3 times/month		1–2 times /week		≥ 3 times/week			*P*_for interaction_ between sex and intake||
	Rarely	HR	95 % CI	HR	95 % CI	HR	95 % CI	*P*_for trend_	Rarely	HR	95 % CI	HR	95 % CI	HR	95 % CI	*P*_for trend_	
Participants, *n*	5046	14 579		9890		5040			5599	17 562		13 746		9340			
Person-years	43 476	127 245		87 526		45 597			50 112	157 300		125 102		87 757			
All causes																	
Deaths, *n*	255	587		469		307			129	264		258		213			
Model 1*	1·00	0·76	0·65, 0·88	0·76	0·65, 0·89	0·78	0·66, 0·92	0·030	1·00	0·64	0·51, 0·79	0·64	0·52, 0·80	0·62	0·50, 0·78	0·003	
Model 2†	1·00	0·79	0·68, 0·91	0·80	0·68, 0·93	0·83	0·70, 0·98	0·128	1·00	0·69	0·55, 0·85	0·71	0·57, 0·89	0·69	0·55, 0·86	0·023	0·238
Model 3‡	1·00	0·81	0·69, 0·94	0·84	0·71, 0·99	0·87	0·73, 1·05	0·391	1·00	0·68	0·55, 0·85	0·72	0·57, 0·90	0·69	0·54, 0·88	0·041	
Model 4§	1·00	0·82	0·70, 0·95	0·87	0·73, 1·03	0·95	0·78, 1·17	0·968	1·00	0·68	0·54, 0·84	0·72	0·57, 0·91	0·76	0·58, 1·00	0·253	
Cancer																	
Deaths, *n*	141	380		286		181			72	169		153		113			
Model 1*	1·00	0·84	0·69, 1·02	0·80	0·65, 0·98	0·80	0·64, 1·01	0·074	1·00	0·70	0·53, 0·92	0·66	0·50, 0·88	0·60	0·44, 0·81	0·004	
Model 2†	1·00	0·86	0·71, 1·05	0·82	0·67, 1·01	0·83	0·66, 1·04	0·130	1·00	0·73	0·55, 0·96	0·71	0·53, 0·94	0·63	0·46, 0·86	0·011	0·167
Model 3‡	1·00	0·86	0·71, 1·05	0·83	0·67, 1·03	0·83	0·66, 1·06	0·161	1·00	0·72	0·54, 0·96	0·71	0·53, 0·96	0·64	0·46, 0·89	0·027	
Model 4§	1·00	0·89	0·73, 1·09	0·88	0·70, 1·10	0·90	0·69, 1·18	0·493	1·00	0·70	0·53, 0·94	0·70	0·51, 0·95	0·69	0·48, 0·98	0·107	
CVD																	
Deaths, *n*	38	67		59		40			20	41		39		36			
Model 1*	1·00	0·62	0·42, 0·93	0·68	0·45, 1·03	0·72	0·46, 1·14	0·374	1·00	0·74	0·43, 1·27	0·67	0·39, 1·17	0·68	0·39, 1·19	0·236	
Model 2†	1·00	0·67	0·45, 1·00	0·75	0·50, 1·14	0·82	0·52, 1·30	0·718	1·00	0·84	0·49, 1·44	0·80	0·46, 1·39	0·79	0·45, 1·39	0·463	0·867
Model 3‡	1·00	0·71	0·47, 1·07	0·87	0·56, 1·34	0·97	0·59, 1·59	0·744	1·00	0·81	0·46, 1·40	0·78	0·44, 1·37	0·78	0·43, 1·42	0·486	
Model 4§	1·00	0·71	0·47, 1·08	0·89	0·56, 1·40	1·11	0·63, 1·93	0·504	1·00	0·76	0·44, 1·33	0·75	0·42, 1·34	0·87	0·45, 1·68	0·785	
Other causes																	
Deaths, *n*	76	140		124		86			37	54		66		64			
Model 1*	1·00	0·66	0·50, 0·88	0·74	0·55, 0·98	0·77	0·56, 1·06	0·387	1·00	0·47	0·31, 0·72	0·59	0·39, 0·88	0·64	0·42, 0·97	0·484	
Model 2†	1·00	0·71	0·54, 0·94	0·80	0·60, 1·07	0·85	0·62, 1·16	0·721	1·00	0·52	0·34, 0·80	0·69	0·46, 1·04	0·75	0·49, 1·14	0·950	0·905
Model 3‡	1·00	0·74	0·56, 0·99	0·87	0·65, 1·18	0·95	0·67, 1·33	0·776	1·00	0·53	0·34, 0·81	0·70	0·46, 1·08	0·75	0·47, 1·19	0·925	
Model 4§	1·00	0·73	0·55, 0·98	0·87	0·63, 1·19	1·03	0·70, 1·51	0·588	1·00	0·55	0·36, 0·85	0·76	0·48, 1·18	0·87	0·52, 1·44	0·759	

CI, confidence interval; CVD, cardiovascular disease; HR, hazard ratio; JDI, Japanese diet index.

* Adjusted for age and study area.

† In males, values are adjusted for covariates in Model 1 plus BMI; smoking habit; alcohol consumption; education level; leisure-time physical activity; and self-reported medical history of hypertension, diabetes and dyslipidaemia.

In females, values are adjusted for covariates in Model 1 plus BMI; smoking habit; alcohol consumption; education level; leisure-time physical activity; self-reported medical history of hypertension, diabetes and dyslipidaemia; age at menarche; number of births; and menopausal status.

‡ Adjusted for covariates in Model 2 plus total energy intake; energy-adjusted intakes of green and yellow vegetables, light-coloured vegetables, fruit, meat, rice, Na and dietary fibre; and JDI score.

§ Adjusted for covariates in Model 3 plus energy-adjusted intakes of *n*-3 HUFA, Ca, vitamin D, retinol and carotene.

|| Adjusted for sex; age; study area; BMI; smoking habit; alcohol consumption; education level; leisure-time physical activity; self-reported medical history of hypertension, diabetes and dyslipidaemia; total energy intake; energy-adjusted intakes of green and yellow vegetables, light-coloured vegetables, fruit, meat, rice, Na and dietary fibre; JDI score; and cross-product term (i.e. sex (dichotomous) × category of small fish consumed (continuous)), representing the interaction.

In the sensitivity analysis additionally excluding participants who died 1–3 years after the baseline survey and using the same covariates as in Model 3, the inverse association between the intake of small fish and all-cause mortality was almost unchanged in females. The HR (95 % CI) were 0·67 (0·53, 0·86) for intakes 1–3 times/month, 0·74 (0·57, 0·94) for 1–2 times/week and 0·67 (0·51, 0·88) for ≥ 3 times/week, compared with the rare intake (*P*_for trend_ = 0·047). No statistically significant trend in the association was observed in males. The HR (95 % CI) were 0·82 (0·69, 0·98) for intakes 1–3 times/month, 0·83 (0·69, 0·99) for 1–2 times/week and 0·90 (0·73, 1·11) for ≥ 3 times/week, compared with the rare intake (*P*_for trend_ = 0·560). In the analysis excluding participants from the Aichi Cancer Center and using the same covariates as in Model 3, the inverse association between the intake of small fish and all-cause mortality was also observed in females. The HR (95 % CI) were 0·71 (0·56, 0·90) for intakes 1–3 times/month, 0·76 (0·59, 0·98) for 1–2 times/week and 0·68 (0·51, 0·89) for ≥ 3 times/week, compared with the rare intake (*P*_for trend_ = 0·041). No statistically significant association was observed in males. The HR (95 % CI) were 0·87 (0·73, 1·04) for intakes 1–3 times/month, 0·91 (0·75, 1·09) for 1–2 times/week and 0·99 (0·81, 1·22) for ≥ 3 times/week, compared with the rare intake (*P*_for trend_ = 0·753). The *P* value for interaction between the intake of small fish and sex for all-cause mortality was not statistically significant (*P*_for interaction_ = 0·238).

### Cause-specific mortality

The associations between the frequency of the intake of small fish and the risk of cancer, CVD and other-cause mortality by sex are also shown in Table 3. In Model 3, the intake of small fish was associated with a statistically significant decrease in the risk of cancer mortality, and a linear trend was observed in females. The multivariable-adjusted HR (95 % CI) were 0·72 (0·54, 0·96) for intakes 1–3 times/month, 0·71 (0·53, 0·96) for 1–2 times/week and 0·64 (0·46, 0·89) for ≥ 3 times/week, compared with the rare intake (*P*_for trend_ = 0·027). The association in males was not statistically significant, although its direction was the same as that in females. In Model 4, with an additional adjustment for the energy-adjusted intake of nutrients abundant in small fish, the inverse association between the intake of small fish and cancer mortality remained statistically significant in females. No statistically significant association was observed in males.

In the sensitivity analysis additionally excluding participants who died 1–3 years after the baseline survey and using the same covariates as in Model 3, the inverse association between the intake of small fish and cancer mortality was almost unchanged in females, whereas no association was detected in males. The HR (95 % CI) in females were 0·77 (0·55, 1·07) for intakes 1–3 times/month, 0·76 (0·54, 1·07) for 1–2 times/week and 0·63 (0·43, 0·93) for ≥ 3 times/week, compared with the rare intake (*P*_for trend_ = 0·039). The corresponding HR (95 % CI) in males were 0·91 (0·72, 1·16), 0·84 (0·65, 1·09) and 0·91 (0·69, 1·22), respectively (*P*_for trend_ = 0·460). In the analysis excluding participants from the Aichi Cancer Center and using the same covariates as in Model 3, the inverse association between the intake of small fish ≥ 3 times/week and cancer mortality was statistically significant in females, but not in males. The HR (95 % CI) in females were 0·79 (0·56, 1·12) for intakes 1–3 times/month, 0·83 (0·58, 1·18) for 1–2 times/week and 0·66 (0·45, 0·98) for ≥ 3 times/week, compared with the rare intake (*P*_for trend_ = 0·081). The corresponding HR (95 % CI) in males were 0·98 (0·76, 1·26), 0·91 (0·70, 1·20) and 1·00 (0·74, 1·34), respectively (*P*_for trend_ = 0·827). The intake of small fish was not clearly associated with the risk of CVD and other-cause mortality in both sexes. The *P* values for interaction between the intake of small fish and sex for the risk were 0·167 for cancer mortality, 0·867 for CVD mortality and 0·905 for other-cause mortality, none of which was statistically significant.

### Stratified analysis

Tables 4 and 5 show the HR (95 % CI) for all-cause and cancer mortality according to the frequency of the intake of small fish in the analyses stratified by age, smoking status and JDI score. In the analysis stratified by age, no remarkable age-dependent differences were found in the directions of the associations between the intake of small fish and all-cause and cancer mortality in both sexes. The *P* values for interaction between the intake of small fish and age for the risk were 0·289 for all-cause mortality and 0·175 for cancer mortality in males. The corresponding *P* values in females were 0·827 for all-cause mortality and 0·909 for cancer mortality. As for the smoking status, the intake of small fish was inversely associated with the risk of all-cause and cancer mortality, especially in never-smoking females. The multivariable-adjusted HR (95 % CI) for all-cause mortality were 0·60 (0·47, 0·76) for intakes 1–3 times/month, 0·62 (0·48, 0·79) for 1–2 times/week and 0·61 (0·47, 0·79) for ≥ 3 times/week, compared with the rare intake (*P*_for trend_ = 0·011). The corresponding HR (95 % CI) for cancer mortality were 0·62 (0·46, 0·85), 0·59 (0·43, 0·82) and 0·53 (0·37, 0·75), respectively (*P*_for trend_ = 0·003). In males, no statistically significant association was observed in either smokers or never smokers. The *P* value for interaction between the intake of small fish and smoking status for the risk was 0·268 for all-cause mortality and 0·059 for cancer mortality in females. The corresponding *P* values in males were 0·892 for all-cause mortality and 0·400 for cancer mortality. Regarding the analysis stratified by the JDI score, the inverse associations between the intake of small fish and the risk of all-cause and cancer mortality were stronger in females with a high JDI score (≥ 4 points). The multivariable-adjusted HR (95 % CI) for all-cause mortality were 0·61 (0·45, 0·83) for intakes 1–3 times/month, 0·63 (0·46, 0·84) for 1–2 times/week and 0·56 (0·41, 0·77) for ≥ 3 times/week, compared with the rare intake (*P*_for trend_ = 0·008). The corresponding HR (95 % CI) for cancer mortality were 0·61 (0·41, 0·93), 0·59 (0·40, 0·89) and 0·52 (0·34, 0·79), respectively (*P*_for trend_ = 0·016). In males, the intake of small fish was inversely associated with the risk of all-cause and cancer mortality in the low JDI score (≤ 3 points) group. The multivariable-adjusted HR (95 % CI) for all-cause mortality were 0·73 (0·60, 0·88) for intakes 1–3 times/month, 0·68 (0·54, 0·86) for 1–2 times/week and 0·84 (0·61, 1·15) for ≥ 3 times/week, compared with the rare intake (*P*_for trend_ = 0·038). The corresponding HR (95 % CI) for cancer mortality were 0·70 (0·55, 0·89), 0·57 (0·42, 0·77) and 0·60 (0·39, 0·91), respectively (*P*_for trend_ < 0·001). Tests of interactions between the intake of small fish and JDI score for all-cause and cancer mortality were statistically significant in males, but not in females. The *P* values for the interaction of all-cause and cancer mortality were 0·045 and 0·002 in males and 0·525 and 0·621 in females, respectively.

**Table 4:** Multivariable-adjusted HR (95 % CI) for all-cause mortality by sex according to the frequency of the intake of small fish in the analysis stratified by age, smoking status and JDI score*

	Male						Female					
	Rarely	1–3 times/month	1–2 times/week	≥ 3 times/week	*P*_for trend_	*P*_for interaction_	Rarely	1–3 times/month	1–2 times/week	≥ 3 times/week	*P*_for trend_	*P*_for interaction_
≥ 60 years old						0·289						0·827
Participants, *n*	1468	4894	4346	3038			1282	4443	5274	5171		
All-cause deaths, *n*	159	369	332	247			71	130	168	164		
HR	1·00	0·78	0·82	0·85	0·467		1·00	0·62	0·71	0·69	0·264	
95 % CI		0·64, 0·94	0·67, 0·99	0·68, 1·05				0·46, 0·83	0·53, 0·96	0·51, 0·94		
< 60 years old												
Participants, *n*	3578	9685	5544	2002			4317	13 119	8472	4169		
All-cause deaths, *n*	96	218	137	60			58	134	90	49		
HR	1·00	0·88	0·90	0·97	0·835		1·00	0·76	0·70	0·65	0·048	
95 % CI		0·69, 1·13	0·68, 1·20	0·68, 1·38				0·55, 1·05	0·49, 0·99	0·42, 0·99		
Smoker (former or current)						0·892						0·268
Participants, *n*	3507	10 346	6981	3465			1177	2894	1691	874		
All-cause deaths, *n*	215	468	373	249			20	56	47	23		
HR	1·00	0·75	0·78	0·86	0·375		1·00	1·21	1·68	1·08	0·515	
95 % CI		0·63, 0·88	0·65, 0·93	0·70, 1·05				0·71, 2·06	0·94, 2·99	0·55, 2·12		
Never smoker												
Participants, *n*	1522	4188	2883	1562			4397	14 577	11 978	8414		
All-cause deaths, *n*	40	118	96	58			106	207	211	187		
HR	1·00	1·10	1·14	0·91	0·657		1·00	0·60	0·62	0·61	0·011	
95 % CI		0·76, 1·58	0·78, 1·68	0·59, 1·41				0·47, 0·76	0·48, 0·79	0·47, 0·79		
JDI score ≤ 3						0·045						0·525
Participants, *n*	3456	8325	3510	906			3820	9880	4845	1422		
All-cause deaths, *n*	167	304	140	59			66	125	78	32		
HR	1·00	0·73	0·68	0·84	0·038		1·00	0·76	0·85	0·99	0·971	
95 % CI		0·60, 0·88	0·54, 0·86	0·61, 1·15				0·56, 1·04	0·60, 1·21	0·63, 1·56		
JDI score ≥ 4												
Participants, *n*	1590	6254	6380	4134			1779	7682	8901	7918		
All-cause deaths, *n*	88	283	329	248			63	139	180	181		
HR	1·00	0·96	1·03	0·98	0·868		1·00	0·61	0·63	0·56	0·008	
95 % CI		0·75, 1·22	0·81, 1·31	0·76, 1·26				0·45, 0·83	0·46, 0·84	0·41, 0·77		

CI, confidence interval; HR, hazard ratio; JDI, Japanese diet index.

* In males, HR were adjusted for age; study area; BMI; smoking habit; alcohol consumption; education level; leisure-time physical activity; self-reported medical history of hypertension, diabetes and dyslipidaemia; total energy intake; energy-adjusted intakes of green and yellow vegetables, light-coloured vegetables, fruit, meat, rice, Na and dietary fibre; and JDI score.

In females, HR were adjusted for the same covariates as in males plus age at menarche, number of births and menopausal status.

**Table 5:** Multivariable-adjusted HR (95 % CI) for cancer mortality by sex according to the frequency of the intake of small fish in the analysis stratified by age, smoking status and JDI score*

	Male						Female					
	Rarely	1–3 times/month	1–2 times/week	≥ 3 times/week	*P*_for trend_	*P*_for interaction_	Rarely	1–3 times/month	1–2 times/week	≥ 3 times/week	*P*_for trend_	*P*_for interaction_
≥ 60 years old						0·175						0·909
Participants, *n*	1468	4894	4346	3038			1282	4443	5274	5171		
Cancer deaths, *n*	85	249	198	144			34	77	95	81		
HR	1·00	0·92	0·86	0·89	0·376		1·00	0·65	0·71	0·61	0·134	
95 % CI		0·72, 1·19	0·66, 1·12	0·66, 1·19				0·43, 0·99	0·47, 1·08	0·40, 0·95		
< 60 years old												
Participants, *n*	3578	9685	5544	2002			4317	13 119	8472	4169		
Cancer deaths, *n*	56	131	88	37			38	92	58	32		
HR	1·00	0·82	0·83	0·82	0·454		1·00	0·78	0·64	0·63	0·057	
95 % CI		0·59, 1·13	0·58, 1·20	0·52, 1·30				0·53, 1·15	0·41, 1·00	0·37, 1·08		
Smoker (former or current)						0·400						0·059
Participants, *n*	3507	10 346	6981	3465			1177	2894	1691	874		
Cancer deaths, *n*	122	303	238	153			10	34	28	16		
HR	1·00	0·78	0·77	0·82	0·264		1·00	1·40	1·81	1·24	0·555	
95 % CI		0·63, 0·97	0·61, 0·98	0·63, 1·07				0·67, 2·91	0·82, 3·99	0·51, 3·04		
Never smoker												
Participants, *n*	1522	4188	2883	1562			4397	14 577	11 978	8414		
Cancer deaths, *n*	19	76	48	28			61	135	125	95		
HR	1·00	1·37	1·10	0·81	0·179		1·00	0·62	0·59	0·53	0·003	
95 % CI		0·82, 2·29	0·63, 1·91	0·43, 1·52				0·46, 0·85	0·43, 0·82	0·37, 0·75		
JDI score ≤ 3						0·002						0·621
Participants, *n*	3456	8325	3510	906			3820	9880	4845	1422		
Cancer deaths, *n*	105	201	82	32			39	86	50	17		
HR	1·00	0·70	0·57	0·60	< 0·001		1·00	0·84	0·83	0·84	0·511	
95 % CI		0·55, 0·89	0·42, 0·77	0·39, 0·91				0·57, 1·25	0·53, 1·30	0·46, 1·54		
JDI score ≥ 4												
Participants, *n*	1590	6254	6380	4134			1779	7682	8901	7918		
Cancer deaths, *n*	36	179	204	149			33	83	103	96		
HR	1·00	1·33	1·34	1·25	0·633		1·00	0·61	0·59	0·52	0·016	
95 % CI		0·92, 1·91	0·93, 1·92	0·86, 1·82				0·41, 0·93	0·40, 0·89	0·34, 0·79		

CI, confidence interval; HR, hazard ratio; JDI, Japanese diet index.

* In males, HR were adjusted for age; study area; BMI; smoking habit; alcohol consumption; education level; leisure-time physical activity; self-reported medical history of hypertension, diabetes and dyslipidaemia; total energy intake; energy-adjusted intakes of green and yellow vegetables, light-coloured vegetables, fruit, meat, rice, Na and dietary fibre; and JDI score.

In females, HR were adjusted for the same covariates as in males plus age at menarche, number of births and menopausal status.

### Intake of small and non-small fish

Supplemental Table 1 summarises the associations between the frequency of the intake of small and non-small fish and the risk of all-cause, cancer, CVD and other-cause mortality by sex in the analysis, adjusting for the intake of small and non-small fish each other in addition to the covariates in Model 3. The intakes of both small and non-small fish were not statistically significantly associated with the risk of each mortality in males. In females, the intake of small fish was statistically significantly associated with a lower risk of all-cause and cancer mortality, but not with CVD and other-cause mortality, even after considering the intake of non-small fish. The multivariable-adjusted HR (95 % CI) for all-cause mortality according to the frequency of the intake of small fish were 0·68 (0·55, 0·85) for intakes 1–3 times/month, 0·72 (0·58, 0·91) for 1–2 times/week and 0·69 (0·54, 0·89) for ≥ 3 times/week, compared with the rare intake (*P*_for trend_ = 0·050). The corresponding HR (95 % CI) for cancer mortality were 0·72 (0·54, 0·96), 0·72 (0·53, 0·97) and 0·65 (0·46, 0·90), respectively (*P*_for trend_ = 0·034). Once a day or more frequent intake of non-small fish was associated with a lower all-cause mortality in females.

## Discussion

In this large prospective study, the frequent intake of small fish was associated with a lower all-cause and cancer mortality in females. The association in males was not statistically significant, although its direction between the intake of small fish and cancer mortality was the same as in females. Regarding the association between the intake of small fish and all-cause mortality in males, the HR were lower for intakes 1–3 times/month or more, compared with the rare intake. The intake of small fish was not associated with CVD mortality in both sexes.

To our knowledge, this is the first study to demonstrate the association between intake of small fish and the risk of all-cause and cause-specific mortality. Small fish can be a component of a healthy diet. They are a good source of micronutrients such as Ca, vitamins and fatty acids when consumed with bones and organs^(2,3,5,6)^. With regard to the relationship between nutrients in small fish and mortality risk, Ca intake is inversely associated with the risk of all-cause and CVD mortality, and some proportion of cancer mortality, such as mortality among patients with early-stage lung cancer^(7,32,33)^; however, some reports suggest that high intake of Ca can increase the risk of cancer and CVD mortality^(7,32,34)^. Thus, appropriate Ca intake, considering individual health status, such as pre-existing diseases (cancer, osteoporosis and heart disease), has been recommended^(7)^. Because Ca from small fish with bones is highly bioavailable, small fish are a useful source of Ca^(5,35)^. Another mechanism for the protective effect of small fish on mortality risk may involve the antitumour effects of vitamins A and D as well as *n*-3 PUFA. Focusing on the major sites associated with cancer death in Japan, recent meta-analyses have shown inverse associations between dietary vitamin A intake and the risk of lung, pancreatic, gastric and breast cancers^(36–40)^. Vitamin D intake or serum 25-hydroxyvitamin D (25(OH)D) level has also been reported to be inversely associated with the risk of lung, breast and colorectal cancer morbidity and mortality^(8,41–44)^. *N*-3 PUFA intake has been related to a reduced risk of breast cancer and is also inversely related to all-cause and CVD mortality in some reports; however, the association is still controversial^(18,45–49)^.

In this study, an association between the intake of small fish and all-cause and cancer mortality was observed in females, and this association remained even after adjustment for female-specific factors, including age at menarche, number of births and menopausal status. One of the reasons underlying the difference in the effects of consumption of small fish on the risk of all-cause and cancer mortality between sexes in this study might be the difference in the cancer type causing cancer mortality among sexes; however, other reasons are unknown. The sex-based difference in the association between the intake of small fish and alcohol and/or other food consumption might provide another explanation although we did consider alcohol drinking and food consumption in the multivariable models. The *P* values for interaction between the intake of small fish and sex for the risk were not statistically significant. The sex difference, if any, could not be so large.

Vitamin A contributes to the prevention of cancer through antioxidant activity, induction of detoxifying enzymes and regulation of genes involved in cell morphogenesis, differentiation and proliferation^(9,37)^. Vitamin D exerts antitumour effects by contact inhibition of proliferation, cell cycle stabilisation, promotion of apoptosis and anti-neoangiogenesis^(8)^. *N*-3 HUFA suppress the progression of carcinogenesis and metastasis through the production of lipid peroxides and increased apoptosis of cancer cells^(50)^. In Model 4 (Table 3), the adjustment for intakes of these nutrients based on the abovementioned findings weakened the inverse association between the intake of small fish and all-cause and cancer mortality. However, the association remained statistically significant in females. This suggests that the effects of these nutrients only partially explain the association of mortality risk, and other or unknown nutrients or physiologically active substances might exert protective effects. Some small fish are rich in Mg^(4)^; however, this was not adjusted for in this study due to the limitation of FFQ. Mg intake reduces the risk of lung cancer^(12)^. Serum Mg is also inversely correlated with all-cause and cancer mortality^(51)^. The intake of small fish is expected to contribute to a well-balanced intake of micronutrients, such as Ca, vitamins A and D, *n*-3 PUFA and Mg.

Participants who consumed higher amounts of small fish could have had a healthier diet as shown in Tables 1 and 2 and demonstrated a lower mortality. To eliminate this confounding factor, we adjusted for the JDI score. The JDI score, which is a measure of the degree of adherence to the Japanese diet, is also an indicator of healthy dietary patterns as, in previous studies, higher JDI scores were reported to be associated with lower risk of all-cause and CVD mortality^(30,31)^. Even after adjustment for the JDI score, the inverse association between the intake of small fish and all-cause and cancer mortality was scarcely altered in females, which suggests that the intake of small fish reduces the risk of all-cause and cancer mortality, independent of a healthy diet. Furthermore, in the analysis adjusting for the intake of non-small fish, the inverse association between the intake of small fish and the risk of all-cause and cancer mortality remained statistically significant in females. This suggests that the intake of small fish is associated with a reduced risk of all-cause and cancer mortality, independent of the intake of non-small fish.

The stratified analysis with respect to the smoking status revealed that the intake of small fish was inversely associated with the risk of all-cause and cancer mortality in female never smokers. We also adjusted for comprehensive smoking variables. These support that confounding by smoking is unlikely. The results of the analysis stratified by the JDI score showed that the inverse association between the intake of small fish and all-cause and cancer mortality was stronger in the high-JDI score group among females. In males, the inverse association was found in the low-JDI score group, but not found in all the males. Although the reason for this sex-based difference is unclear, for males with unhealthy dietary patterns, the intake of small fish may help compensate for the lack of nutrients in poor-quality diets.

The strengths of the present study are the large sample size, prospective design and the extensive adjustment for potentially important confounding factors. Nonetheless, this study has several limitations. First, changes in eating habits or lifestyle factors during the follow-up period could not be considered because the questionnaire was answered only once at the baseline survey by a considerable proportion of participants (40·8 %). Second, the use of an FFQ inevitably led to some misclassification of the intake of small fish, although the questionnaire was validated based on dietary records. The validity was not good for several food groups and nutrients included as covariates. Third, residual confounding, such as socio-economic status, cannot be completely ruled out, although we adjusted for many potential confounding factors. Fourth, the number of CVD death events might not be enough to conclude the association between the intake of small fish and the risk of CVD mortality. Finally, because the study area is limited to Japan, our findings are not generalisable to other countries.

In conclusion, we suggest that the intake of small fish reduces the risk of all-cause and cancer mortality in Japanese females.

## Supporting information

Kasahara et al. supplementary material 1 Kasahara et al. supplementary material

Kasahara et al. supplementary material 2 Kasahara et al. supplementary material

## Supplementary material

For supplementary material accompanying this paper, visit https://doi.org/10.1017/S1368980024000831.
