# Supplementary material for: Association between consumption of small fish and all-cause mortality among Japanese: the Japan Multi-Institutional Collaborative Cohort Study
Source: Public Health Nutr. 2024 May 3;27(1):e135. doi: 10.1017/S1368980024000831 (PMC11148834; doi:10.1017/S1368980024000831)
Supplement: Kasahara et al. supplementary material 1 — Kasahara et al. supplementary material [file S1368980024000831sup001.pdf]

SUPPLEMENTAL TABLE 1 Multivariable-adjusted HR (95% CI) for all-cause, cancer, CVD, and other-cause mortality by sex according to the intake frequency of small and non-small fish, respectively

Intake frequency of small fish	Male					Female				
	Rarely	1–3 times/month	1–2 times/week	≥3 times/week	*P* for trend	Rarely	1–3 times/month	1–2 times/week	≥3 times/week	*P* for trend
Participants, n	5,045	14,562	9,880	5,034		5,579	17,539	13,726	9,322	
All causes										
Deaths, n	255	587	469	306		128	264	258	211	
HR (95% CI)*	1.00	0.80	0.84	0.88	0.427	1.00	0.68	0.72	0.69	0.050
		(0.69–0.94)	(0.71–0.99)	(0.73–1.05)			(0.55–0.85)	(0.58–0.91)	(0.54–0.89)	
Cancer										
Deaths, n	141	380	286	180		71	169	153	112	
HR (95% CI)*	1.00	0.86	0.83	0.85	0.209	1.00	0.72	0.72	0.65	0.034
		(0.71–1.05)	(0.67–1.03)	(0.67–1.08)			(0.54–0.96)	(0.53–0.97)	(0.46–0.90)	
CVD										
Deaths, n	38	67	59	40		20	41	39	36	
HR (95% CI)*	1.00	0.71	0.86	0.96	0.782	1.00	0.80	0.77	0.78	0.513
		(0.47–1.07)	(0.56–1.34)	(0.58–1.57)			(0.46–1.39)	(0.44–1.37)	(0.43–1.44)	
Other causes										
Deaths, n	76	140	124	86		37	54	66	63	
HR (95% CI)*	1.00	0.74	0.87	0.94	0.806	1.00	0.52	0.70	0.74	0.895
		(0.56–0.99)	(0.65–1.18)	(0.67–1.33)			(0.34–0.80)	(0.45–1.08)	(0.47–1.18)	
Intake frequency of non-small fish	Male					Female				
	≤2 times/week	3–4 times/week	5–6 times/week	≥1 time/day	*P* for trend	≤2 times/week	3–4 times/week	5–6 times/week	≥1 time/day	*P* for trend
Participants, n	16,759	11,866	3,391	2,505		19,253	18,197	5,074	3,642	
All causes										
Deaths, n	653	603	174	187		320	340	116	85	
HR (95% CI)†	1.00	1.02	0.85	0.99	0.428	1.00	0.92	0.93	0.75	0.060
		(0.90–1.15)	(0.70–1.02)	(0.82–1.20)			(0.77–1.09)	(0.73–1.19)	(0.57–0.98)	
Cancer										
Deaths, n	410	365	107	105		190	206	68	41	
HR (95% CI)†	1.00	0.95	0.80	0.91	0.184	1.00	0.96	0.97	0.72	0.163
		(0.81–1.12)	(0.63–1.01)	(0.71–1.17)			(0.77–1.20)	(0.71–1.32)	(0.50–1.06)	
CVD										
Deaths, n	75	79	25	25		47	57	13	19	
HR (95% CI)†	1.00	1.17	1.08	1.10	0.753	1.00	0.99	0.64	0.82	0.303
		(0.82–1.68)	(0.65–1.79)	(0.65–1.88)			(0.64–1.52)	(0.33–1.24)	(0.44–1.51)	
Other causes										
Deaths, n	168	159	42	57		83	77	35	25	
HR (95% CI)†	1.00	1.12	0.88	1.15	0.746	1.00	0.80	1.07	0.74	0.489
		(0.87–1.43)	(0.61–1.28)	(0.80–1.63)			(0.56–1.13)	(0.68–1.69)	(0.45–1.24)	

Abbreviations: CI, confidence interval; CVD, cardiovascular disease; HR, hazard ratio; JDI, Japanese diet index.

*In males, HR (95% CI) for all-cause, cancer, CVD and other-cause mortality according to the intake frequency of small fish with adjustments for age; study area; BMI; smoking habits; alcohol consumption; education level; leisure-time physical activity; self-reported medical history of hypertension, diabetes and dyslipidemia; total energy intake; energy-adjusted intakes of green and yellow vegetables, light-colored vegetables, fruit, meat, rice, Na and dietary fiber; JDI score; and the intake frequency of non-small fish.

In females, HR (95% CI) for all-cause, cancer, CVD and other-cause mortality according to the intake frequency of small fish with adjustments for the same covariates as in males plus age at menarche, number of births and menopausal status.

†In males, HR (95% CI) for all-cause, cancer, CVD and other-cause mortality according to the intake frequency of non-small fish with adjustments for age; study area; BMI; smoking habits; alcohol consumption; education level; leisure-time physical activity; self-reported medical history of hypertension, diabetes and dyslipidemia; total energy intake; energy-adjusted intakes of green and yellow vegetables, light-colored vegetables, fruit, meat, rice, Na and dietary fiber; JDI score; and the intake frequency of small fish.

In females, HR (95% CI) for all-cause, cancer, CVD and other-cause mortality according to the intake frequency of non-small fish with adjustments for the same covariates as in males plus age at menarche, number of births and menopausal status.
