# Supplementary material for: Association between consumption of small fish and all-cause mortality among Japanese: the Japan Multi-Institutional Collaborative Cohort Study
Source: Public Health Nutr. 2024 May 3;27(1):e135. doi: 10.1017/S1368980024000831 (PMC11148834; doi:10.1017/S1368980024000831)
Supplement: Kasahara et al. supplementary material 2 — Kasahara et al. supplementary material [file S1368980024000831sup002.pdf]

**SUPPLEMENTAL TABLE 1** Multivariable-adjusted HRs (95% CI) for all-cause, cancer, CVD, and other-cause mortality by sex according to the intake frequency of small and non-small fish, respectively

| Intake frequency of small fish | Male | | | | | Female | | | | |
|---|---|---|---|---|---|---|---|---|---|---|
| | Rarely | 1–3 times/month | 1–2 times/week | ≥3 times/week | *P* for trend | Rarely | 1–3 times/month | 1–2 times/week | ≥3 times/week | *P* for trend |
| Participants, n | 5,045 | 14,562 | 9,880 | 5,034 | | 5,579 | 17,539 | 13,726 | 9,322 | |
| All causes | | | | | | | | | | |
| Deaths, n | 255 | 587 | 469 | 306 | | 128 | 264 | 258 | 211 | |
| HR (95% CI)* | 1.00 | 0.80 | 0.84 | 0.88 | 0.427 | 1.00 | 0.68 | 0.72 | 0.69 | 0.050 |
| | | (0.69–0.94) | (0.71–0.99) | (0.73–1.05) | | | (0.55–0.85) | (0.58–0.91) | (0.54–0.89) | |
| Cancer | | | | | | | | | | |
| Deaths, n | 141 | 380 | 286 | 180 | | 71 | 169 | 153 | 112 | |
| HR (95% CI)* | 1.00 | 0.86 | 0.83 | 0.85 | 0.209 | 1.00 | 0.72 | 0.72 | 0.65 | 0.034 |
| | | (0.71–1.05) | (0.67–1.03) | (0.67–1.08) | | | (0.54–0.96) | (0.53–0.97) | (0.46–0.90) | |
| CVD | | | | | | | | | | |
| Deaths, n | 38 | 67 | 59 | 40 | | 20 | 41 | 39 | 36 | |
| HR (95% CI)* | 1.00 | 0.71 | 0.86 | 0.96 | 0.782 | 1.00 | 0.80 | 0.77 | 0.78 | 0.513 |
| | | (0.47–1.07) | (0.56–1.34) | (0.58–1.57) | | | (0.46–1.39) | (0.44–1.37) | (0.43–1.44) | |
| Other causes | | | | | | | | | | |
| Deaths, n | 76 | 140 | 124 | 86 | | 37 | 54 | 66 | 63 | |
| HR (95% CI)* | 1.00 | 0.74 | 0.87 | 0.94 | 0.806 | 1.00 | 0.52 | 0.70 | 0.74 | 0.895 |
| | | (0.56–0.99) | (0.65–1.18) | (0.67–1.33) | | | (0.34–0.80) | (0.45–1.08) | (0.47–1.18) | |

| Intake frequency of non-small fish | Male | | | | | Female | | | | |
|---|---|---|---|---|---|---|---|---|---|---|
| | ≤2 times/week | 3–4 times/week | 5–6 times/week | ≥1 time/day | *P* for trend | ≤2 times/week | 3–4 times/week | 5–6 times/week | ≥1 time/day | *P* for trend |
| Participants, n | 16,759 | 11,866 | 3,391 | 2,505 | | 19,253 | 18,197 | 5,074 | 3,642 | |
| All causes | | | | | | | | | | |
| Deaths, n | 653 | 603 | 174 | 187 | | 320 | 340 | 116 | 85 | |
| HR (95% CI)† | 1.00 | 1.02 | 0.85 | 0.99 | 0.428 | 1.00 | 0.92 | 0.93 | 0.75 | 0.060 |
| | | (0.90–1.15) | (0.70–1.02) | (0.82–1.20) | | | (0.77–1.09) | (0.73–1.19) | (0.57–0.98) | |
| Cancer | | | | | | | | | | |
| Deaths, n | 410 | 365 | 107 | 105 | | 190 | 206 | 68 | 41 | |
| HR (95% CI)† | 1.00 | 0.95 | 0.80 | 0.91 | 0.184 | 1.00 | 0.96 | 0.97 | 0.72 | 0.163 |
| | | (0.81–1.12) | (0.63–1.01) | (0.71–1.17) | | | (0.77–1.20) | (0.71–1.32) | (0.50–1.06) | |
| CVD | | | | | | | | | | |
| Deaths, n | 75 | 79 | 25 | 25 | | 47 | 57 | 13 | 19 | |
| HR (95% CI)† | 1.00 | 1.17 | 1.08 | 1.10 | 0.753 | 1.00 | 0.99 | 0.64 | 0.82 | 0.303 |
| | | (0.82–1.68) | (0.65–1.79) | (0.65–1.88) | | | (0.64–1.52) | (0.33–1.24) | (0.44–1.51) | |
| Other causes | | | | | | | | | | |
| Deaths, n | 168 | 159 | 42 | 57 | | 83 | 77 | 35 | 25 | |
| HR (95% CI)† | 1.00 | 1.12 | 0.88 | 1.15 | 0.746 | 1.00 | 0.80 | 1.07 | 0.74 | 0.489 |
| | | (0.87–1.43) | (0.61–1.28) | (0.80–1.63) | | | (0.56–1.13) | (0.68–1.69) | (0.45–1.24) | |

Abbreviations: BMI, body mass index; CI, confidence interval; CVD, cardiovascular disease; HR, hazard ratio; JDI, Japanese diet index.

*In males, HRs (95% CI) for all-cause, cancer, CVD, and other-cause mortality according to the intake frequency of small fish with adjustments for age, study area, BMI, smoking habits, alcohol consumption, education level, leisure-time physical activity, self-reported medical history of hypertension, diabetes, and dyslipidemia, total energy intake, energy-adjusted intakes of green and yellow vegetables, light-colored vegetables, fruit, meat, rice, sodium, and dietary fiber, JDI score, and the intake frequency of non-small fish.

In females, HRs (95% CI) for all-cause, cancer, CVD and other-cause mortality according to the intake frequency of small fish with adjustments for the same covariates as in males plus age at menarche, number of births, and menopausal status.

†In males, HRs (95% CI) for all-cause, cancer, CVD, and other-cause mortality according to the intake frequency of non-small fish with adjustments for age, study area, BMI, smoking habits, alcohol consumption, education level, leisure-time physical activity, self-reported medical history of hypertension, diabetes, and dyslipidemia, total energy intake, energy-adjusted intakes of green and yellow vegetables, light-colored vegetables, fruit, meat, rice, sodium, and dietary fiber, JDI score, and the intake frequency of small fish.

In females, HRs (95% CI) for all-cause, cancer, CVD and other-cause mortality according to the intake frequency of non-small fish with adjustments for the same covariates as in males plus age at menarche, number of births, and menopausal status.
